# Hoffa’s Test-Positive Knees Exhibit Increased Stiffness in the Lateral Region of the Infrapatellar Fat Pad: A Shear Wave Elastography Study

**DOI:** 10.7759/cureus.105084

**Published:** 2026-03-12

**Authors:** Yoshinori Komatsu

**Affiliations:** 1 Rehabilitation Science, Sendai Seiyo Gakuin College, Sendai, JPN

**Keywords:** hoffa’s fat pad, hoffa’s test, infrapatellar fat pad, shear wave elastography, tissue stiffness

## Abstract

Objective

This study aimed to quantitatively evaluate the region-specific stiffness of the infrapatellar fat pad (IPFP) using shear wave elastography (SWE) in healthy young adults and to determine whether differences could be detected between individuals with positive and negative results on Hoffa’s test.

Methods

Forty-four healthy young adults participated in this study. IPFP stiffness was measured using SWE with participants in the supine position with the knee fully extended. Measurements were obtained from four regions of the IPFP: subpatellar, central, lateral, and medial. Each region was measured five times, and five regions of interest were set within each area. The mean shear wave velocity (m/s) was calculated and used as the stiffness index. Hoffa’s test was subsequently performed on all participants, who were then classified into Hoffa-positive and Hoffa-negative groups. After confirming normality using the Shapiro-Wilk test, between-group comparisons were conducted using independent samples t-tests. Effect sizes were calculated using Cohen’s d.

Results

Seventeen participants were classified as Hoffa-positive and 27 as Hoffa-negative. The Hoffa-positive group demonstrated significantly greater stiffness in the lateral region of the IPFP than the Hoffa-negative group (p < 0.01, Cohen’s d = 1.31). No significant between-group differences were observed in the other regions.

Conclusions

In healthy young adults, a positive Hoffa’s test was associated with increased stiffness in the lateral region of the IPFP rather than generalized stiffness changes across the entire fat pad. Because this study was cross-sectional and conducted in asymptomatic individuals, these findings should be interpreted as an association rather than as evidence of a causal or pathological relationship. Regional stiffness assessment using SWE may provide quantitative information about localized mechanical characteristics of the IPFP and may complement the conventional clinical examination. Further longitudinal studies, including studies involving symptomatic populations, are required to clarify the clinical relevance of this approach.

## Introduction

The infrapatellar fat pad (IPFP), also known as Hoffa’s fat pad, is a flexible adipose structure located within the knee joint capsule that occupies the anterior compartment of the knee. The IPFP is one of the most highly innervated structures in the knee joint and is therefore highly sensitive to nociceptive stimuli, making it a major source of anterior knee pain [1,2]. For example, in patients with knee osteoarthritis, increased IPFP stiffness is associated with pain severity regardless of the Kellgren-Lawrence grade [3]. Additionally, fibrosis of the IPFP following anterior cruciate ligament reconstruction may reduce elasticity and may contribute to the early development of osteoarthritis [4,5]. Thus, understanding the mechanical properties of the IPFP is important for physical therapy. However, clinical methods for evaluating the IPFP remain limited. Previous MRI studies have shown that abnormalities of the IPFP in individuals with anterior knee pain are often localized, particularly in the superolateral region, rather than being diffusely distributed throughout the entire fat pad [6,7]. These findings suggest that mechanical loading and tissue responses within the IPFP may vary by region.

Hoffa’s test is commonly used for the manual assessment of IPFP disorders. This test involves compressing the IPFP with the fingers while passively extending the knee to provoke pain [8]. Despite its widespread clinical use, it is unclear which specific regions of the anatomically extensive IPFP contribute to a positive result. Furthermore, Hoffa’s test can yield positive results not only in patients with anterior knee pain but also in individuals who do not have clinically evident knee pain, which makes interpretation of its clinical significance more difficult. In particular, no previous studies have quantitatively examined the relationship between Hoffa’s test results and localized tissue characteristics of the IPFP in healthy young adults.

MRI is a useful modality for evaluating the structural and qualitative characteristics of the IPFP; however, its clinical application is limited because of cost and examination time [2,9]. In contrast, shear wave elastography (SWE) is a non-invasive ultrasound technique that enables rapid and quantitative assessment of soft tissue elasticity and has emerged as a promising tool for the visualization and quantification of IPFP tissue properties [10,11]. Previous studies have reported that IPFP stiffness measured using SWE varies depending on the knee flexion angle and the measurement location [12].

In this study, we aimed to quantitatively evaluate the region-specific stiffness of the IPFP using SWE in healthy young adults and to determine whether differences could be detected between individuals with positive and negative Hoffa’s test results. Previous imaging studies have suggested that the lateral region of the IPFP is frequently associated with impingement-related changes and increased mechanical stress related to patellofemoral joint mechanics [6]. Based on this anatomical and imaging evidence, we hypothesized that individuals with a positive Hoffa’s test would demonstrate increased stiffness in the lateral region of the IPFP.

## Materials and methods

Participants

A total of 44 healthy young adults (25 males and 19 females; mean age: 20.3 ± 0.6 years; height: 166.3 ± 8.1 cm; weight: 57.1 ± 8.4 kg) participated in this study. Written informed consent was obtained from all participants before participation. The inclusion and exclusion criteria are summarized in Table 1. The dominant leg, defined as the leg used to kick a ball, was selected for measurement. Because no previous studies had examined the relationship between Hoffa’s test and IPFP stiffness, estimating the required sample size based on an assumed effect size was difficult. Based on preliminary data, the sample size was subsequently re-evaluated to achieve a statistical power of 0.80 with a two-sided significance level of 5%, and additional measurements were performed. This study was conducted in accordance with the principles of the Declaration of Helsinki, and the confidentiality of personal information was ensured. The study protocol was approved by the Research Ethics Committee of Sendai Seiyo Gakuin University and Sendai Seiyo Gakuin College (approval no. 0634).

**Table 1:** Inclusion and exclusion criteria

Category	Criteria
Inclusion criteria	University students aged 18–22 years
	No history of knee trauma or surgery
Exclusion criteria	Limited knee range of motion
	Current knee pain

Shear wave elastography measurement

**Figure 1:**
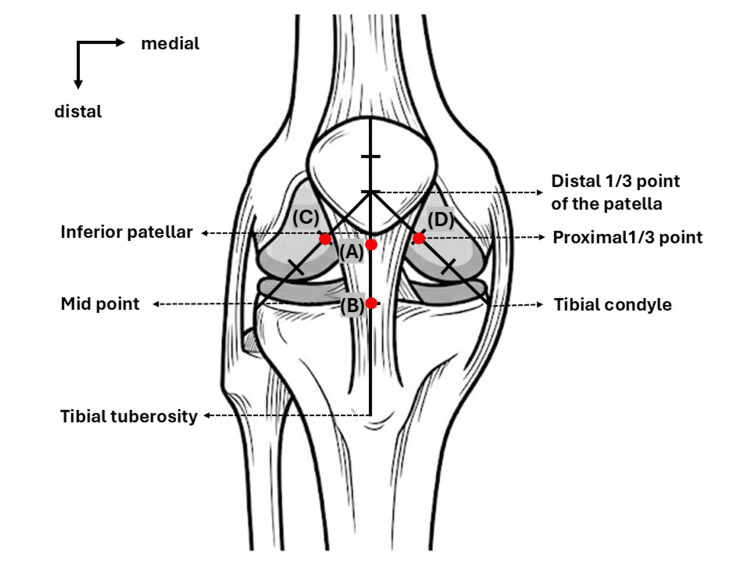
Measurement regions for shear wave elastography of the IPFP (A) Subpatellar region: the portion adjacent to the inferior pole of the patella. (B) Central region: the midpoint of the patellar tendon, defined as the line connecting the inferior pole of the patella and the tibial tuberosity. (C) Lateral region: the proximal one-third point along a line connecting the distal one-third of the patella and the lateral tibial condyle. (D) Medial region: the proximal one-third point along a line connecting the distal one-third of the patella and the medial tibial condyle IPFP: infrapatellar fat pad The figure was created by the author specifically for this manuscript and is an original figure. It has not been previously published and does not require permission from any other source

IPFP stiffness was measured using an ultrasound diagnostic system (Aplio 300; Canon Medical Systems, Tokyo, Japan) equipped with a 10-MHz linear probe in SWE mode. The SWE velocity (m/s) was used as an indicator of the IPFP stiffness [13]. All measurements were performed with participants in the supine position on a platform and the knee positioned at full extension (0°). Before the SWE assessment, the knee range of motion was evaluated using a standard goniometer to confirm the absence of any extension limitation. Measurements were performed while maintaining the knee at 0° extension, and participants were encouraged to remain relaxed during the measurements. Stiffness was assessed in four regions of the IPFP: subpatellar, central, lateral, and medial (Figure 1). The central region was defined as the midpoint of the patellar tendon in the longitudinal plane, in line with previous studies [14]. The subpatellar region was evaluated using the same longitudinal probe orientation as the central region and was defined as the portion of the IPFP adjacent to the inferior pole of the patella [12].

According to previous studies, the lateral and medial regions were located at the proximal one-third point of the line connecting the distal third of the patella and the lateral or medial tibial condyle, respectively [12]. To minimize the influence of probe pressure, the transducer was applied with minimal contact pressure, and a generous amount of ultrasound gel was used to avoid visible tissue compression. Measurements were performed only when stable and homogeneous shear wave propagation maps were clearly confirmed. The SWE measurement box was adjusted to include only the IPFP, while excluding the surrounding tissues. Only measurements with a valid shear wave signal of at least 50% were included in the analysis. Measurements showing obvious signal dropout or unstable color mapping were excluded and repeated.

**Figure 2:**
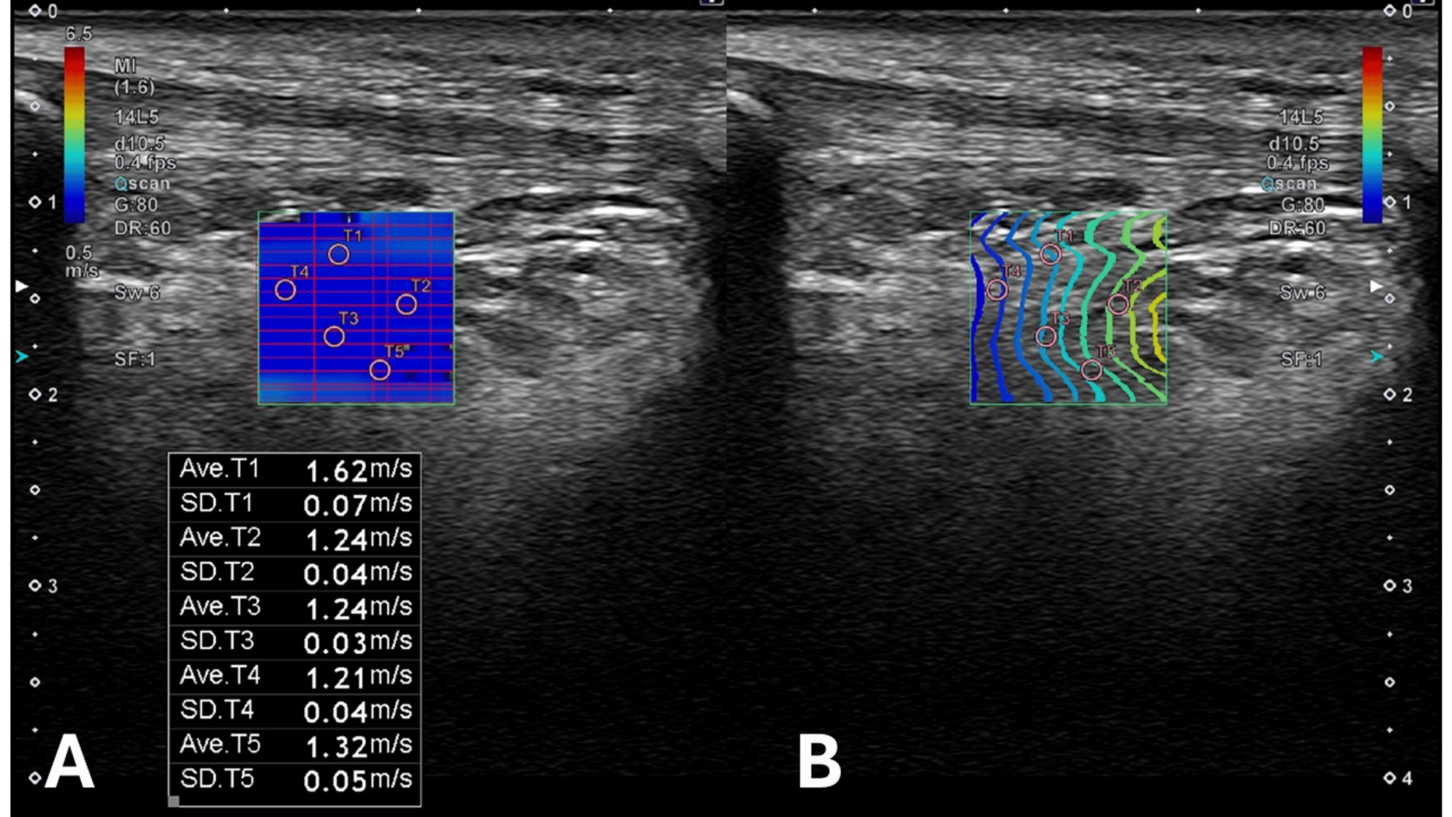
Representative shear wave elastography images of the central region of the IPFP (A) A representative shear-wave velocity map with five circular ROIs placed within the IPFP. (B) The corresponding shear-wave propagation map confirming stable and uniform wave transmission Shear wave velocity (m/s) was used as an indicator of tissue stiffness, and the mean value of the five ROIs was used for analysis. Measurements were performed with the subject in the supine position, with the knee fully extended ROIs: regions of interest; IPFP: infrapatellar fat pad

Each region was measured five times. For each measurement, five circular regions of interest (ROIs) with a diameter of 1 mm were manually placed within the IPFP using a standardized procedure. The ROIs were positioned within the fat pad while avoiding tissue boundaries, bone interfaces, and areas with heterogeneous echogenicity. Homogeneous areas were selected, and the mean value of the five ROIs was used for analysis (Figure 2). All assessments, including Hoffa’s test and SWE measurements, were performed by the same examiner on the same day. The examiner was not blinded to the results of Hoffa’s test at the time of SWE measurement. The IPFP stiffness measurement method used in this study demonstrated good intra-rater reliability (ICC = 0.81) in previous research [12].

Hoffa’s test

Hoffa’s test was performed by compressing the medial and lateral aspects of the IPFP at the inferior margin of the patella with a finger, while passively extending the knee from approximately 30° of flexion [6]. Participants who reported anterior knee pain during the test were classified into the positive group, whereas those without pain were classified into the negative group. Hoffa’s test was performed on the same day as the SWE measurements by the same physical therapist.

Statistical analysis

Normality of the IPFP stiffness data in each region was assessed using the Shapiro-Wilk test. Because all variables showed a normal distribution, independent samples t-tests were used to compare IPFP stiffness between the Hoffa’s test-positive and -negative groups. Homogeneity of variance was examined using Levene’s test, and Welch’s t-test was applied when equal variance could not be assumed. Because four regional comparisons were performed, the significance level was set at p < 0.01 to reduce the risk of type I error. This threshold is more conservative than the Bonferroni-adjusted level for four comparisons (p < 0.0125). Effect sizes were calculated using Cohen’s d to quantify the magnitude of group differences.

Comparisons between regions within each group were not performed, as the primary objective of the study was to examine differences between Hoffa’s test-positive and -negative groups for each region. No formal outlier exclusion was performed, and all data were included in the analysis. Extreme values observed in the distribution were considered to reflect natural biological variability, as all measurements met the predefined quality criteria for SWE signal stability. All statistical analyses were performed using SPSS Statistics version 29.0 (IBM Corp., Armonk, NY).

## Results

Based on Hoffa’s test results, 17 and 27 participants were classified into the positive and negative groups, respectively. The stiffness values of the four IPFP regions for each group are presented in Table 2. An independent samples t-test revealed a significant difference in stiffness in the lateral IPFP region: the Hoffa’s positive group (2.74 ± 0.80 m/s) demonstrated significantly higher stiffness compared with the negative group (1.90 ± 0.51 m/s) (p < 0.01, Cohen’s d = 1.31). No significant differences were observed in the subpatellar, central, or medial regions, and the effect sizes in these regions were small. The distribution of the stiffness values for each IPFP region is illustrated in Figure 2.

**Table 2:** Comparison of IPFP stiffness between Hoffa’s test-positive and -negative groups ^*^P < 0.01 Data were normally distributed. Independent samples t-tests were used for between-group comparisons. Effect sizes were calculated using Cohen’s d IPFP: infrapatellar fat pad; SWE: shear wave elastography; SD: standard deviation; CI: confidence interval

Region (SWE velocity, m/s)	Positive (n = 17), mean ± SD (95% CI)	Negative (n = 27), mean ± SD (95% CI)	P-value	Cohen’s d
Subpatellar	2.23 ± 0.47 (1.99–2.46)	2.01 ± 0.59 (1.71–2.30)	0.27	0.40
Central	2.10 ± 1.56 (1.82–2.38)	1.90 ± 0.42 (1.70–2.11)	0.44	0.41
Lateral	2.74 ± 0.80 (2.24–3.14)	1.90 ± 0.51 (1.64–2.15)	<0.01^*^	1.31
Medial	2.14 ± 0.42 (1.94–2.36)	2.12 ± 0.24 (2.00–2.23)	0.66	0.06

**Figure 3:**
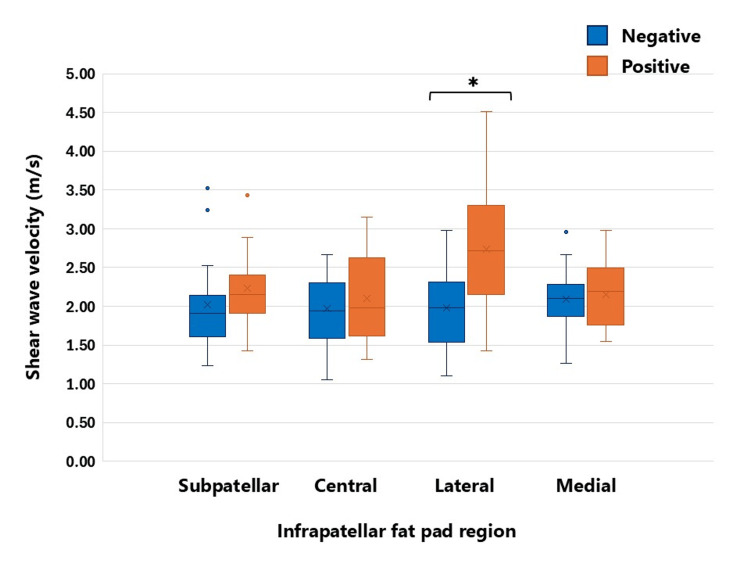
Box-and-whisker plot of IPFP stiffness in Hoffa’s test-positive and -negative groups ^*^P < 0.01 The box-and-whisker plot illustrates IPFP stiffness measured using SWE velocity. Measurements were obtained from the subpatellar, central, lateral, and medial regions of the IPFP. Blue represents the Hoffa’s test-negative group and orange represents the Hoffa’s test-positive group. Boxes indicate the interquartile range; the line within each box represents the median; whiskers indicate the minimum and maximum values, excluding outliers. A significant difference in shear wave velocity between Hoffa’s test-positive and -negative subjects was observed in the lateral region IPFP: infrapatellar fat pad; SWE: shear wave elastography

## Discussion

This study quantified stiffness in four regions (subpatellar, central, lateral, and medial regions) of the IPFP using SWE in healthy young adults and compared this stiffness between Hoffa’s test-positive and Hoffa’s test-negative groups. The results demonstrated significantly higher stiffness in the lateral IPFP region in the Hoffa’s test-positive group, with a large effect size. In contrast, no statistically significant differences were observed in other regions. These findings suggest that a positive Hoffa’s test is associated with localized differences in mechanical properties rather than generalized stiffness changes throughout the IPFP.

The Hoffa’s test is a manual examination designed to provoke anterior impingement-like symptoms by compressing the fat pad from the medial and lateral sides of the patellar tendon while the knee is extended [8]. The lateral IPFP is anatomically positioned in a region that is susceptible to compression by the femoral condyle and the patellofemoral joint during knee extension and has been suggested to experience localized mechanical stress [6]. Previous MRI studies have reported edema and increased T2 signal intensity in the superolateral IPFP in patients with IPFP impingement [7,15].

Furthermore, abnormal patellofemoral alignment or tracking may increase shear forces across the lateral patellofemoral joint, potentially contributing to mechanical compression of the IPFP [6]. Although MRI findings were not evaluated in the present study, the observation that only lateral IPFP stiffness was elevated in the Hoffa’s test-positive group is consistent with the localized changes described in these previous studies. However, because the participants in this study were asymptomatic, healthy individuals, caution is warranted when interpreting whether increased stiffness directly reflects pathological changes.

Despite the absence of knee pain, 38.6% of healthy participants had positive Hoffa’s test results. MRI-based studies have shown that signal abnormalities and edema-like changes in the IPFP are frequently observed not only in patients with knee osteoarthritis or anterior knee pain, but also in asymptomatic individuals, suggesting that imaging findings do not always correlate with pain symptoms [7,16]. The relatively high positive rate may also reflect individual differences in mechanical sensitivity or tissue responsiveness to compression rather than pathological changes. Because Hoffa’s test is a manual examination, the applied compression force may vary depending on the examiner and the participant’s tissue compliance, which could contribute to positive responses in asymptomatic individuals. Therefore, Hoffa’s test positivity in a healthy population should be interpreted as a response to mechanical provocation rather than as an indicator of clinical pathology.

The present findings indicate an association between Hoffa’s test positivity and increased stiffness in the lateral IPFP, suggesting that this clinical test may be sensitive to mechanical alterations in specific IPFP regions rather than abnormalities of the entire fat pad. Clinically, combining Hoffa’s test with a regional stiffness assessment using SWE may provide quantitative information on tissue mechanical properties and complement conventional subjective and qualitative manual examinations. From a clinical perspective, identifying region-specific stiffness characteristics may improve the interpretation of Hoffa’s test findings. While Hoffa’s test can provoke symptoms through fat pad compression, it does not provide information regarding which region of the IPFP may be mechanically involved.

Regional SWE assessment may help clinicians better understand the mechanical basis of a positive test result by identifying localized stiffness alterations. Furthermore, such localized mechanical changes may be present even in asymptomatic individuals and could reflect early mechanical loading conditions rather than established pathology. This information may support clinical decision-making by identifying region-specific mechanical stress and may help guide management strategies aimed at reducing patellofemoral loading or localized lateral IPFP compression. However, the clinical applicability of this approach should be confirmed in future studies involving symptomatic patient populations.

The relationship between IPFP mechanical properties and pain is also an important consideration. Previous studies have reported that increased ultrasound elasticity of the IPFP is associated with anterior knee pain in patients with knee osteoarthritis [3]. However, stiffness in that study was assessed only at a single central location beneath the patellar tendon. In contrast, the present study evaluated multiple regions of the IPFP and demonstrated region-specific mechanical differences associated with Hoffa’s test positivity. Region-based SWE assessment may provide more detailed information regarding localized mechanical alterations within the IPFP and may improve the interpretation of clinical findings when combined with physical examination.

Several limitations of this study should be acknowledged. First, the participants were all healthy young adults, and caution is required when generalizing these findings to clinical populations with anterior knee pain, knee osteoarthritis, or anterior cruciate ligament reconstruction. In addition, knee alignment was not quantitatively assessed in this study. Although participants had no history of knee injury or current symptoms, subtle variations in patellofemoral or lower limb alignment may influence regional mechanical loading of the IPFP and could have affected the regional stiffness findings. Furthermore, because this study used a cross-sectional design, causal relationships between Hoffa’s test findings and regional IPFP stiffness cannot be established. Second, SWE measurements may be influenced by technical factors, such as probe angle and measurement depth.

In addition, all assessments were performed by a single examiner, and the examiner was not blinded to the results of Hoffa’s test at the time of SWE measurement. These factors may have introduced potential measurement bias. Furthermore, inter-rater reliability was not evaluated in the present study. Third, the IPFP is not a static structure, but the morphology and mechanical environment change dynamically during knee motion and muscle contraction [12,17]. Using ultrasound imaging, Hasegawa et al. demonstrated that the IPFP thickness changes during quadriceps contraction, suggesting mechanical interactions between active muscles and the fat pad [17]. Therefore, the IPFP stiffness measured at rest in this study may not directly reflect the mechanical conditions present during movement or pain provocation. In addition, the sample size was relatively modest, which may have limited statistical power and may affect the generalizability of the findings.

Future studies should investigate the relationship between regional IPFP stiffness and clinical symptoms, functional outcomes, and MRI findings in patients with anterior knee pain using a longitudinal study design. In addition, evaluating the inter-rater reliability of SWE-based IPFP stiffness measurements and assessing the mechanical characteristics of the IPFP under dynamic conditions involving muscle activity and joint motion are important. Furthermore, longitudinal evaluation of IPFP stiffness before and after therapeutic interventions may help clarify the clinical relevance and responsiveness of SWE measurements.

## Conclusions

In this study, the regional stiffness of the IPFP was evaluated using SWE in healthy young adults, and comparisons were made between Hoffa’s test-positive and -negative groups. The Hoffa’s test-positive group demonstrated significantly greater stiffness in the lateral IPFP region, whereas no significant differences were observed in the other regions. These findingsHoffa’s test positivity and increased stiffness in the lateral IPFP, rather than a generalized stiffness change across the entire fat pad. Because this study was cross-sectional and conducted in asymptomatic individuals, the results should not be interpreted as indicating a causal or pathological relationship. Regional stiffness assessment using SWE may provide quantitative information on the localized mechanical characteristics of the IPFP and has the potential to complement conventional qualitative clinical examinations, such as Hoffa’s test. Further longitudinal studies, including symptomatic clinical populations, are needed to determine the clinical relevance and diagnostic value of this assessment approach.
